# Cervical digit in a child: A case report

**DOI:** 10.1097/MD.0000000000009348

**Published:** 2017-12-22

**Authors:** Min-Ji Tong, Guang-Heng Xiang, Zi-Li He, Hua-Zi Xu, Nai-Feng Tian

**Affiliations:** Department of Spine Surgery, Zhejiang Spine Research Center, The Second Affiliated Hospital and Yuying Children's Hospital of Wenzhou Medical University, Wenzhou, Zhejiang, China.

**Keywords:** cervical spine, congenital abnormalities, digit-like bone, ossification of the nuchal ligament

## Abstract

**Background:**

A “digit-like” bone is a rare developmental anomaly that is usually seen in the pelvic or thoracic regions. Such an anomaly in the cervical spine is extremely rare and few cases have been reported. We present a patient with an anomalous bone posterior to a cervical vertebra. The patient was admitted to our hospital with a gradually growing hard neck mass and esthetic complaints. Physical examination, radiographs, reconstructed computed tomography, and magnetic resonance imaging revealed a digit-like bone posterior to the cervical spine. The patient was diagnosed with a “cervical digit.” Through a posterior midline approach, the anomalous bone was excised because of its gradually increasing size and esthetic complaints.

**Results:**

Intraoperatively, the bony mass was found to have a pseudoarticulation with the spinous process of C5 (the fifth cervical vertebra). The specimen consisted of normal bone and cartilage. The child returned to a normal life postoperatively with no symptoms. There was no recurrence at the 2-year follow-up.

**Conclusion:**

A congenital cervical digit is a rare deformity. A detailed clinical workup and advanced imaging examinations are useful for diagnosing such conditions. Esthetic complaints contribute to surgical indications. This is the first cervical digit managed with surgical excision of the anomalous bone and had a favorable outcome.

## 1 Introduction

Congenital abnormalities of the cervical spine are not uncommon. Most patients are asymptomatic and diagnosed incidentally during radiological examinations. Therefore, the true incidence of congenital cervical anomalies is unknown and is likely largely underestimated.^[1]^ In comparison to relatively common deformities, a congenital anomalous bone formation on the cervical spine, also called a “cervical digit,” is extremely rare. A “digit-like” bone is a developmental anomaly that is usually seen in the pelvic or thoracic region. No intervention is required for majority of the cases. Previously, a neonate with a digit-like bone attached to the cervical spine has been reported.^[2]^ Here, we present a patient with a similar anomaly in childhood. The differential diagnosis and embryological development of the anomaly are also discussed.

## 2 Case report

**Figure 1:**
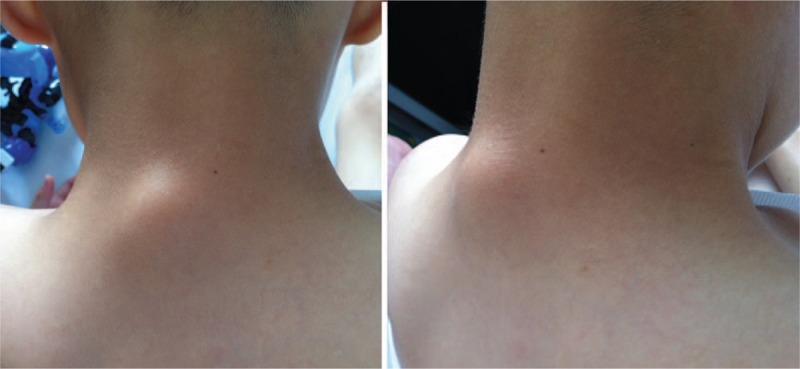
Photographs of the patient showed the posterior cervical protuberance.

A 6-year-old boy presented to our hospital with a hard neck mass. He did not complain of pain or discomfort in the neck. His parents stated that the mass was observed immediately after he was born and it had grown gradually with age, especially in the past 2 years. He had never been treated before for the mass. He had no history of trauma or persistent fever. His mother's pregnancy and delivery history were normal and the boy exhibited normal growth and development at 6 years of age, with no relevant family history. Physical examination revealed a hard, immobile mass posterior to the cervical spine (Fig. 1). There was no swelling or tenderness of the mass. The mass was oval, measuring 3 × 1 cm. The skin temperature was normal and the skin was not attached to the mass. There was no sign of local compression of nerves, arteries, or veins. The patient had full cervical range of motion and the mass was more evident with flexion. No masses were detected elsewhere in his body. His neurological examination was unremarkable. No pain was evoked during the physical examination. All laboratory findings were normal.

**Figure 2:**
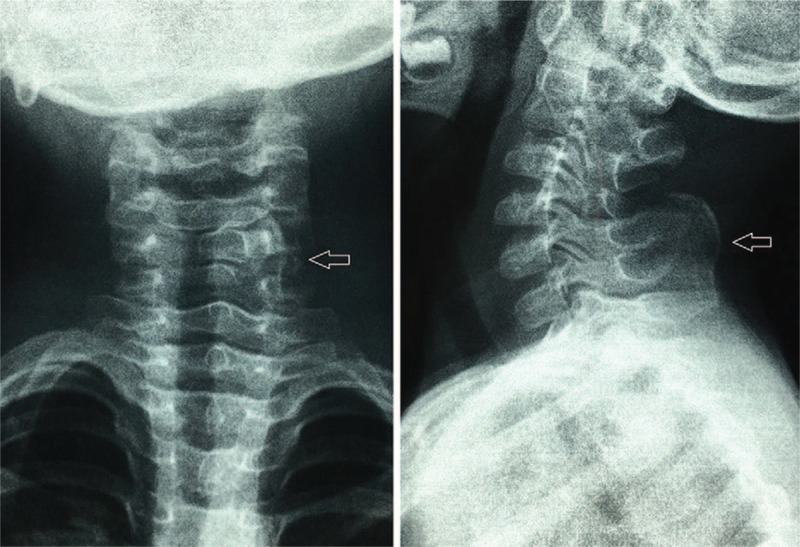
Plain radiographs of the cervical spine demonstrated an anomalous bone located posterior to the C5 vertebrae (arrow).

**Figure 3:**
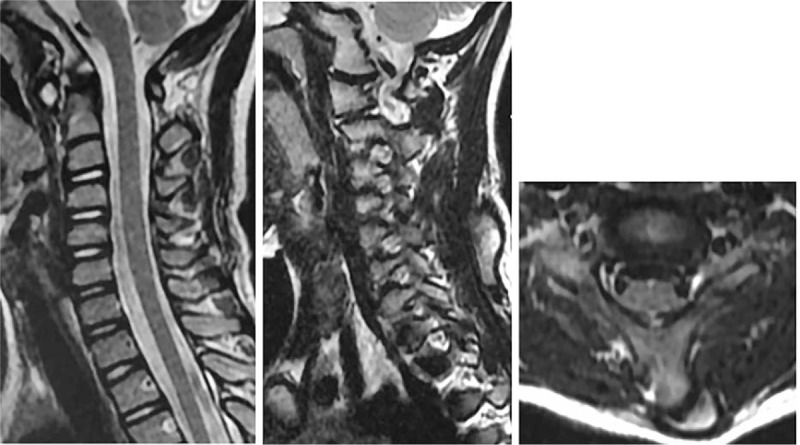
MRI scans showed normal spinal cord and intervertebral discs. MRI also revealed a well-circumscribed cortical bone within the dorsal cervical muscles. The abnormal bone was connected to the spinous process of C5 with cartilage tissue. MRI = magnetic resonance imaging.

**Figure 4:**
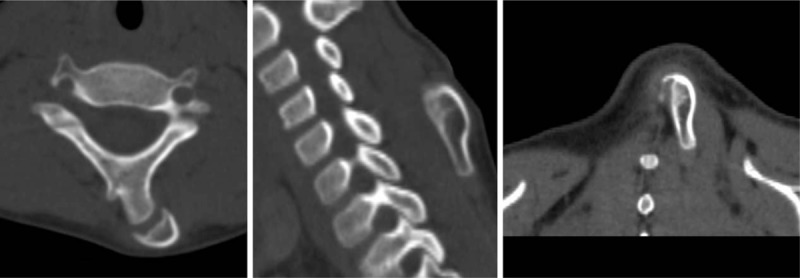
The reconstructed CT scans showed the well corticated anomalous bone articulating with the spinous process of C5. CT = computed tomography.

**Figure 5:**
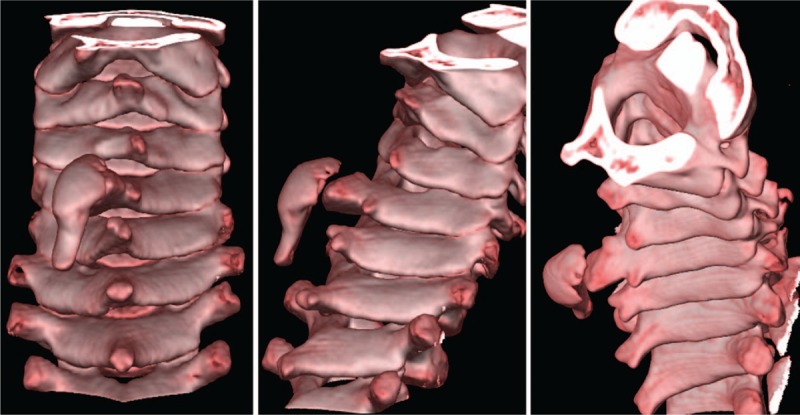
Three dimensional CT scans revealed a left side finger-like growth attached to the spinous process of the C5 vertebra. The spinous process of the C5 vertebra was hypoplastic. CT = computed tomography.

Plain radiographs (Fig. 2) showed an anomalous bone attached to the left side of the C5 (the fifth cervical) vertebra. Magnetic resonance imaging of the cervical spine showed a normal spinal cord and intervertebral discs (Fig. 3). It also revealed a well-circumscribed cortical bone within the dorsal cervical muscles (Fig. 3). Two- and 3-dimensional computed tomography revealed a finger-like bone with a pseudoarticulation attached to the hypoplastic spinous process of the left side of the C5 vertebra (Figs. 4 and 5). The anomalous bone had an obvious cortex and medulla osseous structure (Fig. 4).

**Figure 6:**
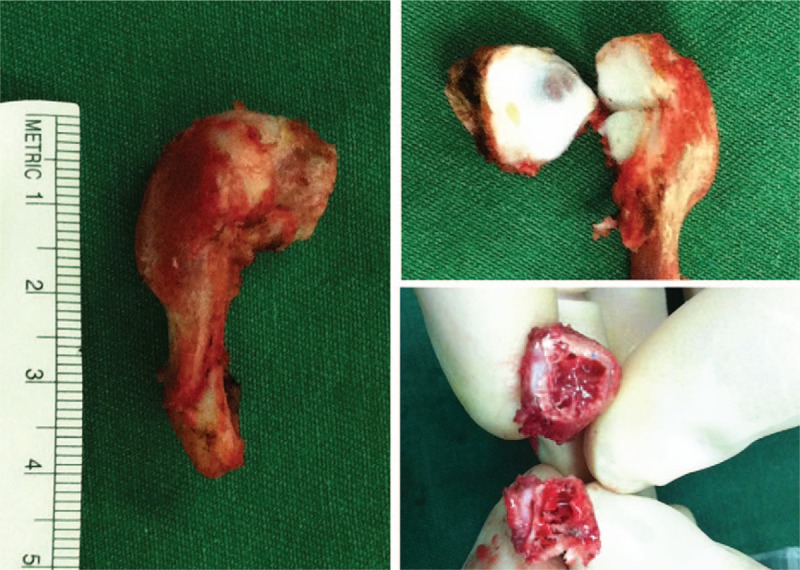
Intraoperative photographs of the gross specimen revealed the anomalous bone with clear cortex and medulla as well as a pseudoarticulation base.

The patient was diagnosed with a “cervical digit.” Because of its gradually increasing size and esthetic complaints, the patient underwent surgical excision of the anomalous bone trough a posterior midline approach. Intraoperatively, the bony mass was found to have a pseudoarticulation with the spinous process of C5. No obvious structure was found to connect the bony mass to the surrounding soft tissue. The bone was removed together with the pseudoarticulation and part of the hyperplastic spinous process of C5 (Fig. 6). Histological examination showed that the specimen consisted of normal bone and cartilage. The postoperative course was uneventful and the boy returned to a normal life. At the 2-year follow-up, he was symptom free and there was no recurrence. This study was approved by the ethics committee of The Second Affiliated Hospital and Yuying Children's Hospital of Wenzhou Medical University and the informed consent was obtained from the patient.

## 3 Discussion

This condition was deemed a cervical digit based on the imaging and surgical findings. A digit-like bone is a rare congenital anomaly in which bone develops in soft tissue adjacent to normal skeletal bone. It is usually seen in the pelvic or thoracic region and is rarely observed in the cervical spine. The typical radiographic finding is a rib- or digit-like bone with a clear cortex and medulla attached to normal bone with a characteristic pseudoarticulation at its base.^[3]^ One hypothesis to explain this deformity is the migration of embryonic mesoderm with rib-forming capacity to an atypical location.^[4]^ Another theory is that the anomaly arises in the mesenchymal stage of bone growth within the first 6 weeks of embryogenesis, when independent cartilaginous costal primordium fuses with the vertebral column in all except the thoracic vertebrae. Fusion failure causes the cartilaginous center to keep growing and form a supernumerary rib.^[5]^

A digit-like bone with a cervical vertebra is extremely rare. A literature review revealed 5 case reports.^[2,6–9]^ Moreover, some of these cases might not be true cervical digits because of the lack of detailed information regarding the differential diagnosis, which includes posttraumatic myositis ossificans, avulsion fractures, and heterotopic bone formation, which have been described in detail elsewhere.^[10–12]^ We also think that ossification of the nuchal ligament (ONL) should be included in the differential diagnosis in the cervical region, especially for adults. ONL is an acquired osseous structure with a patchy, irregular shape. It results, frequently, from chronic overload of the nuchal ligament. The prevalence of ONL is 23% in healthy adults,^[13]^ and reaches 49.7% in patients with cervical spondylosis.^[14]^ High blood glucose, high body mass index, advanced age, and male sex are associated with ONL.^[13–16]^ Furthermore, the location of ONL correlates with the level of osteophyte formation, cervical disc degeneration, foramen narrowing, and spinal canal stenosis.^[15,16]^ Of note, 4 of the 5 case reports had some of the characteristics of ONL, including middle-aged or older men, midline location, irregular shape, and corresponding level degeneration. Therefore, a firm diagnosis for these cases requires more detailed information. In comparison, Nakano's case^[2]^ and our patient had unique features. The anomalies in both cases were observed immediately after birth, indicating a congenital anomaly. The ectopic bone deviated to 1 side rather than the midline where the nuchal ligament was located. The growing anomaly in our case raised an esthetic problem, contributing to the surgical indication, which has never been reported. The surgical specimen consisted of ectopic bone with a clear cortex and medulla, as well as a pseudoarticulation at its base, which confirmed the diagnosis of a cervical digit.

This study has certain limitations. The surgical indication for a cervical digit is controversial as the growth potential of the anomaly is currently unknown. It is unclear whether growth of the anomalous bone would interfere with normal structure and function of the cervical spine. In our case, surgery was performed because of rapid growth of the mass and the appearance of the abnormality. It is unknown whether the treatment we undertook was optimal in this case. Nevertheless, the surgical outcome was favorable; therefore, our report could guide future clinical decisions in similar cases.

In conclusion, congenital cervical digit is a rare deformity. Detailed clinical examination and advanced imaging are useful for diagnosis in such cases. Ossification of the nuchal ligament is an important differential diagnosis to consider, especially in adult patients. The increased size of the mass and local aesthetic abnormality is often the surgical indication. Furthermore, surgical excision of the anomalous bone in our case resulted in a favorable outcome.
